# Association between self-reported sleep apnea and albuminuria among middle age and elderly population: Results from National Health and Nutrition Examination Survey

**DOI:** 10.1097/MD.0000000000041515

**Published:** 2025-02-07

**Authors:** Li-Da Chen, Xiao-Fen Lin, Qiao-Zhen Xu, Zhi-Ming Cai, Ji-Guang Zhou, Li Lin, Xiao-Bin Zhang

**Affiliations:** a Department of Respiratory and Critical Care Medicine, Zhangzhou Affiliated Hospital of Fujian Medical University, Zhangzhou, Fujian Province, China; b Department of Geriatric Medicine, Zhangzhou Affiliated Hospital of Fujian Medical University, Zhangzhou, Fujian Province, China; c Department of Information Technology, Zhangzhou Affiliated Hospital of Fujian Medical University, Zhangzhou, Fujian Province, China; d Department of Pulmonary and Critical Care Medicine, Zhongshan Hospital, Xiamen University, Xiamen, Fujian Province, China; e The School of Clinical Medicine, Fujian Medical University, Fuzhou, Fujian Province, China.

**Keywords:** albuminuria, elderly, NHANES, renal damage, sleep apnea

## Abstract

The prevalence of both obstructive sleep apnea (OSA) and albuminuria increase with advanced age. The data on the association between OSA and albuminuria in subjects with advanced age were limited. Hence, the present study aimed to assess the association between sleep apnea (SA) and albuminuria in middle age and elderly population. Data on participants with age ≥ 40 years during 2005 to 2008 and 2015 to 2020 National Health and Nutrition Examination Survey were analyzed. SA was evaluated based on the sleep questionnaire and albuminuria was assessed by albumin-to-creatinine ratio. The independent relationship between SA and albuminuria was explored by using multivariate logistic regression. A total of 13,902 subjects with 11,788 cases of normoalbuminuria and 2114 cases of albuminuria were included for data analysis. The proportion of albuminuria increased as SA severity aggravation. Univariate logistic analysis showed that frequently SA was positively associated with albuminuria (OR = 1.301, 95% CI = 1.089–1.547, *P* = .003). In multivariate logistic analysis, frequently SA was independently associated with increased risk of albuminuria after adjusting for confounding factors (OR = 1.309, 95% CI = 1.010–1.683, *P* = .039). The present study suggested that self-reported frequently SA was independently associated with increased risk of albuminuria in middle age and elderly population.

## 1. Introduction

Obstructive sleep apnea (OSA) is a common sleep disorder characterized by intermittent upper-airway collapse, which disrupts sleep and impairs ventilation. The prevalence of OSA in the overall population ranges from 9% to 38% based on apnea-hypopnea index (AHI) ≥ 5 events/h, with 90% in elderly men and 78% in elderly women. Based on AHI ≥ 15 events/h, the prevalence of OSA in the general adult population ranges from 6% to 17%, being as high as 49% in the advanced ages.^[1]^ Accumulating evidence from epidemiologic and laboratory studies demonstrated the strong correlations between OSA and increased risk of cardiovascular disease,^[2]^ diabetes,^[3]^ chronic kidney disease (CKD),^[4]^ and neurocognitive impairment.^[5]^ OSA is emerging as a major health problem due to its both high prevalence and serious complications.

Albuminuria is an indicator of early renal damage and high albuminuria is associated with increased risk of end-stage renal disease, acute kidney injury, progressive CKD, and even death.^[6,7]^ The prevalence of microalbuminuria in general populations ranges from 4.6% to 7%.^[8–10]^ Older age is a well-known independent risk factor for albuminuria and the prevalence of albuminuria increases with age.^[11–13]^ A few studies have investigated the association between OSA and albuminuria.^[14,15]^ Considering that the prevalence of both OSA and albuminuria increase with advanced ages, the effect of OSA on albuminuria in subjects with advanced ages is worth for further investigations. In addition, the data on this issue is limited.

Hence, the present study aimed to assess the association between sleep apnea (SA) and albuminuria in middle age and elderly population based on data from the National Health and Nutrition Examination Survey (NHANES) 2005-2008, 2015-2020 collection cycles.

## 2. Methods

### 2.1. Data source

NHANES is a series of ongoing cross-sectional surveys conducted by the Centers for Disease Control and Prevention. The dataset can be downloaded and analyzed from the NHANES website (https://www.cdc.gov/nchs/nhanes/index.html), and is accessible free of any costs. The National Center for Health Statistics Research Ethics Review Board approved the NHANES protocol and all participants provided informed written consent. The NHANES data is available to the public after anonymization. NHANES data sets from 2009 to 2014 did not include sleep related questionnaire (code: SLQ040), so we excluded these data sets. A cross-sectional, retrospective cohort analysis of the 2005 to 2008 and 2015 to 2020 NHANES database was performed in this study.

### 2.2. Study population

The participants of 40 years old or older with complete questionnaires data on sleep disorders (snoring, gasping, and stopping breathing while sleeping) and complete urine albumin and creatinine assessments were selected for our research. We further excluded pregnant women and subjects self-reported that a doctor had told them they had kidney problems.

### 2.3. Outcome assessment

Random spot urine samples were collected in sterile cups and frozen. Urinary albumin was measured with a solid-phase fluorescent immunoassay and urinary creatinine was tested using modified Jaffe kinetic method or enzymatic method. Albumin-to-creatinine ratio (ACR) was calculated as follows: ACR (mg/g) = urine albumin (mg/L)/urine creatinine (g/L). Albuminuria was as defined as a urine ACR ≥ 30 mg/g. The urine ACR < 30 mg/g was considered to be normoalbuminuria.

### 2.4. Exposure assessment

SA was evaluated based on the sleep questionnaire (code: SLQ040) as follows: how often did you snort, gasp, or stop breathing while asleep? The presence of SA depended on the subject’s answers to this sleep questionnaire: never, rarely (1 to 2 nights per week), occasionally (3 to 4 nights per week), and frequently (5 or more nights per week). A frequency of rarely, occasionally, or frequently was considered to have SA. Furthermore, severity of SA was assessed based on it.

### 2.5. Covariate assessment

Sex (males or females), age (years), body mass index (BMI), race (Mexican American, other Hispanic, non-Hispanic White, non-Hispanic Black, other race), smoking status, drinking, hypertension, diabetes, and coronary heart disease (CHD) were selected as the potential confounding variables. The BMI was measured in the mobile examination center and calculated using the formula: BMI = weight (kg)/height (m^2^). A history of hypertension, diabetes, CHD was defined based on a self-reported physician diagnosis of hypertension, diabetes, CHD. There are 3 categories of smoking status: never, former, current. We further combined the classifications of smoking: smoking: Yes (current), No (former, never). The drinking status included drinking and non-drinking.

### 2.6. Statistical analysis

The continuous variables were presented as mean (standard deviation) or medians (interquartile range) and categorical variables were presented as numbers and percentages. The differences between 2 groups were analyzed using the Mann–Whitney U-test for skewed variables and Student *t* test for normally distributed variables. For comparisons between more than 2 groups, 1-way analysis of variance (ANOVA) was used for normally distributed variables, while the Kruskal–Wallis test was applied for skewed variables. The categorical variables were compared by the Chi-squared test. Univariate and multivariable logistic regression analyses were conducted to evaluate the association between SA and albuminuria. Gender, age, BMI, and race were adjusted in model 1. Gender, age, BMI, race, smoking, and drinking were adjusted in model 2. Gender, age, BMI, race, smoking, drinking, diabetes, hypertension, and CHD were adjusted in model 3. All *P*-values were 2 sided, and values <.05 were considered statistically significant. All analyses were performed using R software (version 4.3.0; R Foundation for Statistical Computing).

## 3. Results

**Figure 1.**
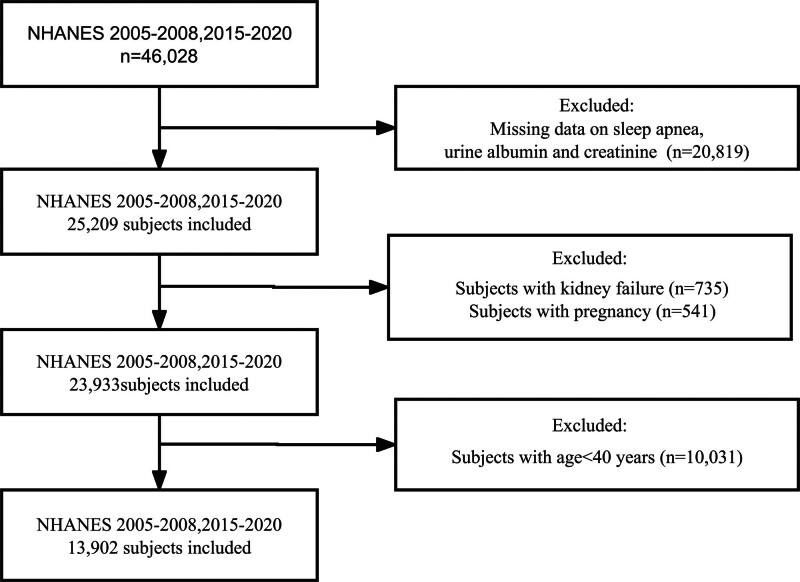
The flow chart of selection process.

Initially, a total of 46,028 subjects were identified. After excluding the missing data on questionnaire of SA and laboratory data of urine, 25,209 participants were further included. We further excluded subjects with kidney failure (n = 735), pregnancy (n = 541), age <40 years (n = 10,031). Finally, 13,902 participants were enrolled in the present analysis (Fig. 1).

Anthropometric and SA parameters of all subjects stratified by ACR are summarized in Table 1. 11,788 and 2114 subjects were categorized into normoalbuminuria and albuminuria group, respectively. Subjects with albuminuria tended to be older, have higher BMI, and exhibit higher prevalence of hypertension, diabetes, CHD, smoking, while having a lower prevalence of drinking. No significant difference of sex ratio or smoking rate existed between albuminuria and normoalbuminuria groups. The proportion of race and SA status significantly varied between 2 groups. In addition, the anthropometric characteristics and albuminuria status of all subjects stratified by SA severity were summarized in Table 2. There were no significant differences in the racial distribution and smoking rates between the 4 groups. The age, BMI, and proportion of male gender, hypertension, diabetes, CHD, drinking, albuminuria significantly increased as SA severity aggravation.

**Table 1:** Anthropometric and SA parameters of all subjects stratified by ACR.

	Normoalbuminuria	Albuminuria	*P*-values
Number of subjects	11,788	2114	
Sex			
Male, number (%)	5853 (49.7)	1086 (51.4)	.152
Female, number (%)	5935 (50.3)	1028 (48.6)	
Age, yr	58.00 (48.00–68.00)	65.00 (54.00–76.00)	<.001
BMI, kg/m^2^	28.50 (25.00–32.79)	29.17 (25.00–34.20)	<.001
Race			
Mexican American, number (%)	1621 (13.8)	319 (15.1)	.001
Other Hispanic, number (%)	1204 (10.2)	197 (9.3)	
Non-Hispanic White, number(%)	5063 (43.0)	830 (39.3)	
Non-Hispanic Black, number(%)	2622 (22.2)	541 (25.6)	
Other Race, number (%)	1278 (10.8)	227 (10.7)	
Hypertension			
Yes, number (%)	5016 (42.6)	1350 (64.1)	<.001
No, number (%)	6763 (57.4)	757 (35.9)	
Diabetes			
Yes, number (%)	1639 (13.9)	789 (37.4)	<.001
No, number (%)	10,142 (86.1)	1323 (62.6)	
CHD			
Yes, number (%)	563 (4.8)	203 (9.7)	<.001
No, number (%)	11,183 (95.2)	1886 (90.3)	
Smoking			
Yes, number(%)	2105 (38.2)	427 (40.1)	.242
No, number(%)	3410 (61.8)	637 (59.9)	
Drinking			
Yes, number (%)	7509 (68.0)	1215 (62.4)	<.001
No, number (%)	3535 (32.0)	732 (37.6)	
SA status			
No SA, number (%)	8794 (74.6)	1561 (73.8)	.001
Rarely SA, number (%)	1387 (11.8)	210 (9.9)	
Occasionally SA, number (%)	871 (7.4)	173 (8.2)	
Frequently SA, number (%)	736 (6.2)	170 (8.0)	

Skewed data (age and BMI) were expressed as median (interquartile range). Categorical variables were presented as number (percentage).

ACR = albumin-to-urine creatinine ratio, BMI = body mass index, CHD = coronary heart disease, SA = sleep apnea.

**Table 2:** Anthropometric characteristics and albuminuria status of all subjects stratified by SA severity.

	No SA	Rarely SA	Occasionally OSA	Frequently SA	*P*-values
Number of subjects	10,355	1597	1044	906	
Sex					
Male, number (%)	4854 (46.9)	911 (57.0)	621 (59.5)	553 (61.0)	<.001
Female, number (%)	5501 (53.1)	686 (43.0)	423 (40.5)	353 (39.0)	
Age, yr	60.00 (49.00–70.00)	58.00 (49.00–67.00)	59.00 (50.00–68.00)	57.00 (48.00–66.00)	<.001
BMI, kg/m^2^	28.05 (24.60–32.15)	29.30 (25.80–34.10)	29.80 (26.20–34.60)	32.24 (27.99–37.51)	<.001
Race					
Mexican American, number (%)	1470 (14.2)	219 (13.7)	127 (12.2)	124 (13.7)	.174
Other Hispanic, number (%)	1012 (9.8)	168 (10.5)	109 (10.4)	112 (12.4)	
Non-Hispanic White, number (%)	4373 (42.2)	689 (43.1)	435 (41.7)	396 (43.7)	
Non-Hispanic Black, number (%)	2362 (22.8)	365 (22.9)	254 (24.3)	182 (20.1)	
Other Race, number (%)	1138 (11.0)	156 (9.8)	119 (11.4)	92 (10.2)	
Hypertension					
Yes, number(%)	4526 (43.8)	784 (49.2)	543 (52.1)	513 (56.6)	<.001
No, number(%)	5817 (56.2)	811 (50.8)	499 (47.9)	393 (43.4)	
Diabetes					
Yes, number (%)	1679 (16.2)	307 (19.2)	226 (21.6)	216 (23.9)	<.001
No, number (%)	8668 (83.8)	1290 (80.8)	818 (78.4)	689 (76.1)	
CHD					
Yes, number(%)	513 (5.0)	92 (5.8)	79 (7.6)	82 (9.1)	<.001
No, number(%)	9787 (95.0)	1501 (94.2)	962 (92.4)	819 (90.9)	
Smoking					
Yes, number(%)	1815 (38.1)	294 (37.2)	208 (40.7)	215 (41.4)	.291
No, number(%)	2944 (61.9)	496 (62.8)	303 (59.3)	304 (58.6)	
Drinking					
Yes, number (%)	6330 (65.6)	1100 (73.1)	697 (71.1)	597 (69.2)	<.001
No, number (%)	3313 (34.4)	405 (26.9)	283 (28.9)	266 (30.8)	
Albuminuria					
Yes, number (%)	1561 (15.1)	210 (13.1)	173 (16.6)	170 (18.8)	.001
No, number (%)	8794 (84.9)	1387 (86.9)	871 (83.4)	736 (81.2)	

Skewed data (age and BMI) were expressed as median (interquartile range). Categorical variables were presented as number (percentage).

BMI = body mass index, CHD = coronary heart disease, OSA = obstructive sleep apnea, SA = sleep apnea.

Table 3 describes the results of univariate logistic analysis for albuminuria. The univariate logistic analysis revealed that age, BMI, the presence of hypertension, diabetes, CHD, frequently SA were positively associated with albuminuria. While the race of Non-Hispanic White, drinking, and presence of rarely SA were negatively correlated with albuminuria. There was no relationship between smoking, gender and albuminuria.

**Table 3:** The results of univariate logistic analysis for albuminuria.

	Albuminuria	*P*-values
	OR (95% CI)	
Sex		
Female	1	
Male	1.071 (0.976–1.175)	.145
Age	1.039 (1.035–1.043)	<.001
BMI	1.017 (1.010–1.023)	<.001
Race		
Mexican American	1	
Other Hispanic	0.831 (0.685–1.007)	.060
Non-Hispanic White	0.833 (0.724–0.960)	.011
Non-Hispanic Black	1.048 (0.902–1.221)	.541
Other Race	0.903 (0.749–1.086)	.278
Hypertension		
No	1	
Yes	2.404 (2.185–2.648)	<.001
Diabetes		
No	1	
Yes	3.690 (3.330–4.088)	<.001
CHD		
No	1	
Yes	2.138 (1.804–2.524)	<.001
Smoking		
No	1	
Yes	1.086 (0.949–1.241)	.228
Drinking		
No	1	
Yes	0.781 (0.707–0.864)	<.001
SA status		
No SA	1	
Rarely SA	0.853 (0.729–0.994)	.044
Occasionally SA	1.119 (0.940–1.325)	.200
Frequently SA	1.301 (1.089–1.547)	.003

BMI = body mass index, CHD = coronary heart disease, SA = sleep apnea, OR = odds ratio.

We further performed a multivariate logistic regression to evaluate the independent relationship between SA and albuminuria (Table 4). Model adjusting for gender, age, BMI, and race revealed that frequently SA had an increased risk of albuminuria (OR = 1.336, 95% CI = 1.109–1.603, *P* = .002). After adjusting for gender, age, BMI, and race, smoking and drinking, the effect of frequently SA on albuminuria was still significant (OR = 1.395, 95% CI = 1.086–1.778, *P* = .008). In addition, after adjusting for gender, age, BMI, race, smoking, drinking, diabetes, hypertension, and CHD, we still determined that the frequently SA group had a significantly higher OR (1.309, 95% CI = 1.010–1.683, *P* = .039). Finally, a dose–response association was observed between the severity of SA and high-risk of albuminuria (test for trend, *P* = .013).

**Table 4:** The multivariate logistic analysis for relationship between SA and albuminuria.

SA status	Model 1		Model 2		Model 3	
	OR (95% CI)	*P*–values	OR (95% CI)	*P*–values	OR (95% CI)	*P*–values
No SA	1		1		1	
Rarely SA	0.865 (0.735–1.013)	.076	0.923 (0.732–1.154)	.489	0.858 (0.676–1.080)	.199
Occasionally SA	1.100 (0.918–1.311)	.294	1.341 (1.045–1.708)	.019	1.251 (0.966–1.607)	.084
Frequently SA	1.336 (1.109–1.603)	.002	1.395 (1.086–1.778)	.008	1.309 (1.010–1.683)	.039
*P*-value for trend		<.001		<.001		.013

Model 1: adjusted for gender, age, BMI, and race, Model 2: adjusted for gender, age, BMI, race, smoking, and drinking, Model 3: adjusted for gender, age, BMI, race, smoking, drinking, diabetes, hypertension, and CHD.

BMI = body mass index, CHD = coronary heart disease, OR = odds ratio, SA = sleep apnea.

## 4. Discussion

This large retrospective study of community populations with advanced ages showed that the presence of frequently SA was significantly associated with increased risk of albuminuria, independent of gender, age, BMI, race, smoking, drinking, diabetes, hypertension, and CHD. The finding suggested that the frequently SA was a risk factor in the progression of early renal damage.

Epidemiological evidences support a bi-directional relationship between OSA and CKD. OSA is fairly common in adult CKD populations.^[4,16]^ A recent study revealed that 31% of patients in the early stage of CKD and 45% in the late stage were found to have OSA.^[16]^ A meta-analysis reported a prevalence of SA of 57% in individuals with CKD after pooling the data of 32 single studies. Furthermore, a pooled analysis of 91 studies found a prevalence of 49% in the end-stage kidney disease population.^[17]^ These findings underscore the high burden of OSA in kidney disease populations. The high prevalence of OSA is not explained solely by increasing BMI, aging or comorbid disease, suggesting that the kidney disease itself plays a role.^[18,19]^ The impact of OSA on CKD was also demonstrated in emerging evidence. A multicenter cross-sectional study including 1295 adults reported that moderate and severe OSA had an increased risk of CKD progression independent of other CKD risk factors.^[20]^ Another retrospective study including nonobese patients with CKD found that nocturnal hypoxia was an independent risk factor of a rapid decline in kidney function.^[21]^

Several studies assessed the relationship between OSA and early markers of renal damages. Bulcun et al^[22]^ compared 98 OSA patients and 26 nonapneic subjects and revealed that ACR were higher in OSA group than control group. Further linear regression model indicated a negative relationship between ACR and minimal O_2_ and significant positive relationship between ACR and desaturation index. Another study involving 273 adults with type 2 diabetes mellitus found a significant and independent correlation between the severity of OSA and albuminuria, even after adjusting for other factors.^[15]^ However, the previous studies were limited by small sample size and hospital-based population. In addition, the data on solely advanced age patients were limited.

The community-based nature and large-sample size were strengths of the present study. In this study, we demonstrated a significant independent relationship between frequently SA and increased risk of albuminuria in middle-aged and older adults. Our findings have some potential clinical implications. Firstly, selecting early renal damage as our research outcome is beneficial for early treatment and leads to a better prognosis. Secondly, identifying modifiable risk factors for early renal damage is also vital for prevention. Considering that OSA can be effectively treated by continuous positive airway pressure (CPAP), it is promising to use this to reduce the risk of progression of renal damage. A previous meta-analysis including 6 articles and 211 subjects has suggested that CPAP therapy resulted in a favorable effect on the decrease of ACR in subjects with OSA.^[23]^ Finally, our current study suggests that the simple sleep questionnaire is feasible in identifying the risk factor of early renal damage.

Mechanisms by which OSA may result in kidney damage include intermittent hypoxia, sympathetic nervous system activation, hypertension, diabetes, and activation of the renin–angiotensin system (RAS).^[4,24]^ Robust evidences demonstrated that OSA could lead to hypertension,^[25]^ diabetes,^[3]^ sympathetic nervous system activation,^[26]^ inflammation, oxidative stress,^[27]^ and activation of the RAS.^[28]^

There were some limitations in this clinical study. Firstly, it was a cross-sectional study, which did not allow for causal analysis. Secondly, the sleep measures did not include polysomnography to characterize SA. We only had a screen for symptoms of SA based on sleep questionnaire. The reliance on a self-reported questionnaire may result in underdiagnosis or misclassification. However, snoring, gasping, or stopping breathing while sleeping could most likely be a sign of SA if the person knows or is told it. Future studies with more reliable diagnostic tools are necessary to further validate these findings. Thirdly, our study could not exclude all confounding factors, which might impact the results. Fourthly, we only included subjects with age ≥ 40 years. Thus, results cannot be generalized to the young adult or pediatric population. Finally, albuminuria was estimated only once in this study. While a single measurement of albuminuria is commonly used in clinical practice, false positive results could occur due to factors such as urinary tract infections. Ideally, albuminuria should be measured at least 3 times to reduce the possibility of false positives and provide more accurate assessments of renal function.

In summary, the present large-sample cross-sectional study demonstrated that self-reported frequently SA was independently associated with increased risk of albuminuria in middle age and elderly population. The result supports the importance of screening and treatment of OSA in middle age and elderly patients with early renal damages.

## Acknowledgments

We would like to thank all participants in this study.

## Author contributions

**Conceptualization:** Xiao-Bin Zhang.

**Data curation:** Li-Da Chen, Xiao-Bin Zhang.

**Formal analysis:** Xiao-Fen Lin, Xiao-Bin Zhang.

**Investigation:** Li-Da Chen, Xiao-Bin Zhang.

**Methodology:** Li-Da Chen, Qiao-Zhen Xu.

**Project administration:** Xiao-Bin Zhang.

**Software:** Ji-Guang Zhou.

**Writing – original draft:** Li-Da Chen.

**Writing – review & editing:** Zhi-Ming Cai, Li Lin, Xiao-Bin Zhang.
